# Statistics of Anæsthesia

**Published:** 1891-09

**Authors:** 


### Statistics of Anæsthetics.

We believe that St. Bartholomew's Hospital is the only one in the metropolis in which a record is kept, and statistics published of the number of times anæsthetics are administered during the year. These statistics are both interesting and instructive, and become valuable as showing the direction of the current in favor of or against a particular anæsthetic agent. Turning, for example, to the records of 1879, we find that out of 2,094 anæsthetizations chloroform was given 975 times, nitrous oxide gas alone 112 times, ether alone 23 times, and ether preceded by nitrous oxide 984 times. In 1889, however, the records are as follows: In 3,606 administrations—chloroform, 1,601 times, gas 686, ether 810, gas and ether 509 times. Thus, on comparing these figures the remarkable fact becomes apparent that chloroform has again come to the front as the most popular anæsthetic at St. Bartholomew's Hospital. Not only does the mixture of ether and gas not maintain its position of superiority as was the case in the year 1879, but in 1889 not even the total administrations of ether alone, and gas and ether combined reach by a long way the number of administrations of chloroform. It is just possible that in part this change of opinion may be due to the results published by the Hyderabad Commission, of which a member of the staff of the hospital was the shining light. But investigation shows that ether has been decling in favor for some years at St. Bartholomew's. In 1888 it was administered 1,003 times out of 3,788 ; while, during the same year, gas and ether combined was only given 349 times. What a contrast this with eleven years ago, as the records above quoted demonstrate! As we see now, chloroform takes the lead, then a long way behind comes ether alone, while gas and ether combined make a shocking bad third.—*Medical Press and Circular.*

